# Chimeric Antigen Receptor–Modified T Cells and T Cell–Engaging Bispecific Antibodies: Different Tools for the Same Job

**DOI:** 10.1007/s11899-021-00628-2

**Published:** 2021-04-30

**Authors:** Melanie Schwerdtfeger, Mohamed-Reda Benmebarek, Stefan Endres, Marion Subklewe, Vincenzo Desiderio, Sebastian Kobold

**Affiliations:** 1 Center of Integrated Protein Science Munich (CIPS-M) and Division of Clinical Pharmacology, Department of Medicine IV, Klinikum der Universität München, LMU Munich, Munich, Germany; 2 Department of Experimental Medicine, University of Campania “Luigi Vanvitelli”, Naples, Italy; 3 German Center for Translational Cancer Research (DKTK), Munich, Germany; 4 Einheit für Klinische Pharmakologie (EKLiP), Helmholtz Zentrum München, German Research Center for Environmental Health (HMGU), Neuherberg, Germany; 5 Department of Medicine III, Klinikum der Universität München, LMU Munich, Munich, Germany

**Keywords:** Chimeric antigen receptor, Bispecific antibody, Immunotherapy, Adoptive T cell therapy, T cell redirection, Cancer

## Abstract

**Purpose of Review:**

Both chimeric antigen receptor (CAR) T cells and T cell–engaging antibodies (BiAb) have been approved for the treatment of hematological malignancies. However, despite targeting the same antigen, they represent very different classes of therapeutics, each with its distinct advantages and drawbacks. In this review, we compare BiAb and CAR T cells with regard to their mechanism of action, manufacturing, and clinical application. In addition, we present novel strategies to overcome limitations of either approach and to combine the best of both worlds.

**Recent Findings:**

By now there are multiple approaches combining the advantages of BiAb and CAR T cells. A major area of research is the application of both formats for solid tumor entities. This includes improving the infiltration of T cells into the tumor, counteracting immunosuppression in the tumor microenvironment, targeting antigen heterogeneity, and limiting off-tumor on-target effects.

**Summary:**

BiAb come with the major advantage of being an off-the-shelf product and are more controllable because of their half-life. They have also been reported to induce less frequent and less severe adverse events. CAR T cells in turn demonstrate superior response rates, have the potential for long-term persistence, and can be additionally genetically modified to overcome some of their limitations, e.g., to make them more controllable.

## Introduction

In efforts to harness T cells in the fight against cancer, several immunotherapeutic approaches have been successfully developed. Among others, chimeric antigen receptor (CAR) T cells and T cell–engaging bispecific antibodies (BiAb) have gained approval by regulatory agencies and are currently being used to treat patients with hematological malignancies.

Both BiAb and CAR T cells use antibodies or antibody fragments to redirect T cells to specific tumor-associated antigens, which is a shared facet of these major histocompatibility complex (MHC)–independent approaches. Their clinical application has achieved unprecedented response rates in patients with relapsed or refractory B cell malignancies, although in only partially overlapping indications [1, 2]. Both can induce severe adverse events like cytokine release syndrome (CRS) and neurotoxicity. Further, a large proportion of patients inevitably relapse, and the efficacy of BiAb or CAR T cells targeting solid tumors remains limited [3••].

BiAb are recombinant proteins with antigen-binding antibody domains both for T cell–specific and tumor-associated antigens. When infused into the patient, they can redirect endogenous T cells to kill cancer cells expressing a specific target [4].

CAR T cells are usually generated by genetically modifying patient-derived T cells ex vivo before their adoptive transfer back into the patient. A CAR is a synthetic receptor consisting of a single-chain variable fragment (scFv) linked to a transmembrane domain and intracellular T cell–activating domains. CAR binding to the antigen on the tumor cell surface activates the CAR T cell and triggers a T cell response against antigen-expressing tumor cells [5•].

In this review, we present and describe different formats of BiAb and CAR T cell therapies. We compare BiAb with CAR T cells, highlighting the differences and similarities, as well as the advantages and limitations of either strategy. In line with this, we outline preclinical and clinical strategies that are currently in development to overcome therapeutic limitations and boost efficacy.

## T Cell–Engaging Bispecific Antibodies

The term BiAb will be used in this review for all antibody-based molecules containing antigen-binding sites for both T cell and tumor-associated antigens. Generally, BiAb can be divided into BiAb containing an Fc domain and Ab fragment–based ones. Labrijn et al. provide an extensive overview of the different BiAb formats [6•].

Most BiAb with an Fc domain bear mutations introduced to abolish Fc-mediated effector functions such as antibody-dependent cellular cytotoxicity, phagocytosis, and complement-dependent cytotoxicity, given that they can result in off-target immune cell activation [6•, 7, 8]. However, these BiAb are usually designed to maintain binding of the neonatal Fc receptor (FcRn) which protects them from degradation, thus conferring a long plasma half-life (days) compared to the plasma half-life of fragment-based BiAb (hours) [9–13]. This can be advantageous as they can be administered in a bolus injection, whereas fragment-based BiAb need to be infused continuously. The drawback is that they are more slowly eliminated from the circulation in the occurrence of adverse events. Fragment-based BiAb can be produced relatively easily at high yields and low costs but are more prone to aggregation or stability issues [14]. Generally, they exhibit faster tissue penetration than Fc-containing BiAb, including crossing of the blood-brain barrier. This distinction is a double-edged sword, as it may increase patient susceptibility to neurotoxicity, while being more favorable for the treatment of brain tumors [15•]. The opposite applies to larger BiAb with an Fc domain, which are actively exported from the brain by transcytosis mediated by FcRn [9].

BiAb valency, i.e., the number of binding arms, as well as the affinity of the individual binding domains can greatly influence the functionality and biodistribution of a BiAb. In the case of a CD3-binding BiAb, one binding site for CD3 is preferred to prevent unwanted T cell activation by CD3 cross-linking [2••]. A reduced affinity for CD3 can minimize BiAb trapping in tissues containing a high number of T cells [6•, 16, 17]. In addition, BiAb with reduced potency can be administered at higher doses to augment efficacy while limiting adverse events. In contrast, two tumor antigen–binding domains can increase selective recognition and killing of highly antigen-expressing tumor cells by increasing the avidity (through the simultaneous binding of both arms) while sparing healthy cells expressing the antigen at lower levels [7, 18–20]. In addition, lowering the affinity for both the CD3 and tumor antigen–binding domains have also been shown to widen the therapeutic window [21].

In 2009, the first BiAb was approved by the European Medicines Agency (EMA). Although more than 40 BiAb are currently in phase 1 and 2 clinical trials for both hematological and solid cancers, to date only two molecules have gained regulatory approval for cancer therapy [22]. Removab® (catumaxomab), an anti-CD3 × anti-epithelial cellular adhesion molecule (EpCAM) BiAb containing an Fc domain, was intraperitoneally applied to treat malignant ascites in ovarian cancer but was withdrawn from the market in 2017 for commercial reasons.

Blincyto® (blinatumomab), an anti-CD3 × anti-CD19 fragment–based bispecific T cell engager (BiTE®), is the only BiAb currently on the market. It gained approval for B cell precursor acute lymphoblastic leukemia (ALL) by the US Food and Drug Administration (FDA) in 2014 and by the European Medicines Agency in 2015. Lacking an Fc domain, and thus not protected from degradation by FcRn, it has a half-life of approximately 1 to 2 h and can therefore only be administered via a continuous intravenous infusion [10, 11]. Complete response rates ranged from 36 to 69% in clinical trials (see Table 1).

**Table 1:** Comparison between CAR T cells and BiAb

	CAR T cells	BiAb
Structure	T cells genetically engineered to express a synthetic receptor consisting of an extracellular scFv linked to intracellular activation and co-stimulatory domains	Recombinant soluble protein with binding domains for a T cell and a tumor antigen
Signals for T cell activation	Signal 1 (CD3ζ), signal 2 (CD28, 4-1BB; in 2nd and 3rd generation CAR constructs), signal 3 (cytokine stimulation ex vivo)	Signal 1 (CD3ζ)
Immune synapse	Atypical [37]	Classical [36]
Effector cells	Engineered CD8^+^ and CD4^+^ T cells; less differentiated T cells show better efficacy in vivo	Endogenous CD8^+^ and CD4^+^ T cells; mainly antigen-experienced T cells kill
Manufacturing	Autologous CAR T cells: individual production for each patient	Off-the-shelf product
	Allogeneic CAR T cells: production in batches from healthy donor T cells (investigational use only)	
	Prone to manufacturing variability (T cell subset composition, transduction efficiency, number of viable T cells) and failure	
Pre-treatment	Lymphocyte apheresis for collecting T cells (for autologous T cells), lymphodepletion chemotherapy before CAR T cell infusion	Dexamethasone to limit CRS and neurotoxicity
Dosing	Single dose	Multiple dosing, for short half-life formats continuous infusion
Costs	Up to 320,000 € in Germany [63]	Up to 293,000 € in Germany [64]
Regulatory approval	Kymriah: r/r B cell precursor ALL patients up to 25 years (FDA 2017, EMA 2018), adult patients with large B cell lymphoma (FDA and EMA 2018) [25, 27]	Blinatumomab: r/r B cell precursor ALL (FDA 2014, EMA 2015 (only Philadelphia chromosome–negative ALL)), B cell precursor ALL with minimal residual disease (FDA 2018, EMA 2019 (only adults)) [65, 66]
	Yescarta: adult patients with large B cell lymphoma (FDA 2017, EMA 2018) [26, 28]	
	Tecartus: adult patients with r/r mantle cell lymphoma (FDA and EMA 2020) [29, 30]	
Complete response rates (CR/CRh/CRi)	Adult B cell ALL: 83 to 93% [67–69]	Adult B cell ALL: 36 to 69% [76–80]
	Pediatric B cell ALL: 70 to 94% [70–73]	
	Diffuse large B cell lymphoma: 40 to 57% [52, 53, 74, 75]	
	Mantle cell lymphoma: 67% [31]	
Relapse rates (% of complete responders)	Adult B cell ALL: 12 to 61% [68, 69]	Adult B cell ALL: 40 to 70% [76–78, 80]
	Pediatric B cell ALL: 26 to 40% [70–72]	
	Diffuse large B cell lymphoma: 21% [75]	
CD19-negative relapse (% of all relapses)	B cell ALL: 16 to 68% [69–72]	Adult B cell ALL: 8 to 30% [76, 81]
Toxicities	More frequent and severe CRS (≥ grade 3: 13 to 47%) and neurotoxicity (≥ grade 3: 5 to 50%), on-tumor off-target effects (B cell aplasia when targeting CD19) [52, 53, 68–71, 73, 75]	CRS (≥ grade 3: 2 to 6%) and neurotoxicity (≥ grade 3: 7 to 17%), on-tumor off-target effects (B cell aplasia when targeting CD19) [76–80]

Other BiAb currently under clinical investigation include, e.g., BiTE molecules targeting CD20 in chronic lymphoblastic leukemia, CD33 in acute myeloid leukemia, and B cell maturation antigen (BCMA) in multiple myeloma [15•].

Beyond BiAb, CAR T cells comprise a promising arm of cancer immunotherapy which is introduced in the next section.

## CAR T Cells

CAR structure typically consists of an extracellular antigen recognition domain (in most cases an antibody-derived scFv) connected via a spacer and a transmembrane domain to one or more intracellular signaling domains [23•]. These domains determine the type of signal transmitted after the scFv engages its target. While first generation CAR constructs can only propagate signal 1 via the intracellular CD3ζ chain of the TCR complex, second-generation CAR constructs have an additional co-stimulatory domain, in most cases the intracellular domain of CD28 or 4-1BB, through which signal 2 is transmitted. In third-generation CAR constructs, two co-stimulatory domains are included, further augmenting the co-stimulus.

Individual CAR features can greatly impact CAR T cell function, including T cell phenotype, persistence, tonic signaling, and on-target off-tumor effects. For example, lowering the affinity of the scFv can help CAR T cells discern tumor cells differentially expressing the antigen from healthy cells expressing it at lower levels, thus limiting on-target off-tumor responses [24]. In addition, exchanging the co-stimulatory domain has been shown to impact T cell activation as well as the in vivo persistence of CAR T cells (as observed when swapping the CD28 co-stimulus for 4-1BB) [23•]. Also, the transduction of specific T cell subsets, the method of transgene delivery, and selection of the promoter can influence the efficacy and adverse effects of CAR T cells [1, 23•]. This topic has recently been reviewed in more detail elsewhere [23•].

After clinical trials showed dramatic response rates, two CAR T cell products targeting the B cell antigen CD19 received marketing authorization by the FDA in 2017 and the EMA in 2018 for relapsed or refractory (r/r) B cell malignancies after two or more lines of systemic treatment [25–28]. Kymriah (tisagenlecleucel) is approved for r/r B cell precursor ALL and large B cell lymphoma, and Yescarta (axicabtagene ciloleucel) for large B cell lymphoma. Both use second-generation CAR constructs but differ in their co-stimulatory domains: 4-1BB for Kymriah and CD28 for Yescarta. Complete response rates in ALL range from 70 to 94% but are lower in diffuse large B cell lymphoma with 40 to 57% (see Table 1).

In addition, Tecartus (brexucabtagene autoleucel) has been approved in 2020 by the FDA and EMA for r/r mantle cell lymphoma [29, 30]. It utilizes the same anti-CD19 CAR as Yescarta and achieved a complete response in 67% of patients in the clinical trial that led to its regulatory approval [31].

More than 200 CAR T cell products are currently being evaluated in clinical trials for a variety of different targets in both hematological and solid malignancies, with more than 40 trials started in 2020 alone [32, 33]. For example, anti-BCMA CAR T cells have shown promising results in multiple myeloma patients and are currently under regulatory review [34]. Most studies use patient-derived autologous T cells, while a minority uses allogeneic T cells from healthy donors. Allogeneic T cells on the one hand hold the promise of a standardized off-the-shelf product with lower costs and the added option for repeated infusions. On the other hand, they need to include additional genetic modifications to lower the risk of graft-versus-host disease and alloimmunization [3••].

There is certainly more to come from CAR T cells as anti-cancer therapeutics. This growing potential, and how it compares to that of BiAb therapy, are outlined below.

## Comparison of CAR T Cells and BiAb

Both CAR and BiAb approaches are distinctly advantageous in their own right. Although a clinical trial comparing these approaches within the same cohort for the same indication is still lacking, it remains important to compare and contrast these approaches. This is what we aim to outline in this section, highlighting differences in their mode of action, manufacturing, and clinical applications.

### Signals Provided for T Cell Activation

Optimal T cell activation requires three signals: signal 1 is normally provided by the T cell receptor (TCR)-major histocompatibility complex (MHC) interaction, signal 2 through a co-stimulatory receptor on the T cells binding its ligand on antigen-presenting cells or target cells, and signal 3 by cytokines such as interleukin (IL)-2, IL-7, and IL-15 [3••, 35]. CAR activation itself provides signal 1 through the CD3ζ intracellular domain and signal 2 through the co-stimulatory domains. BiAb only provide signal 1 by activating the CD3 receptor [3••, 35]. As CAR T cells are stimulated with cytokines during manufacturing, thereby providing signal 3, they have an additional advantage regarding T cell activation [35]. This may contribute to the fact that, based on the currently approved products, CAR T cells are considered more efficacious than blinatumomab (see Table 1).

### Immune Synapses and Killing Mechanisms

**Fig. 1:**
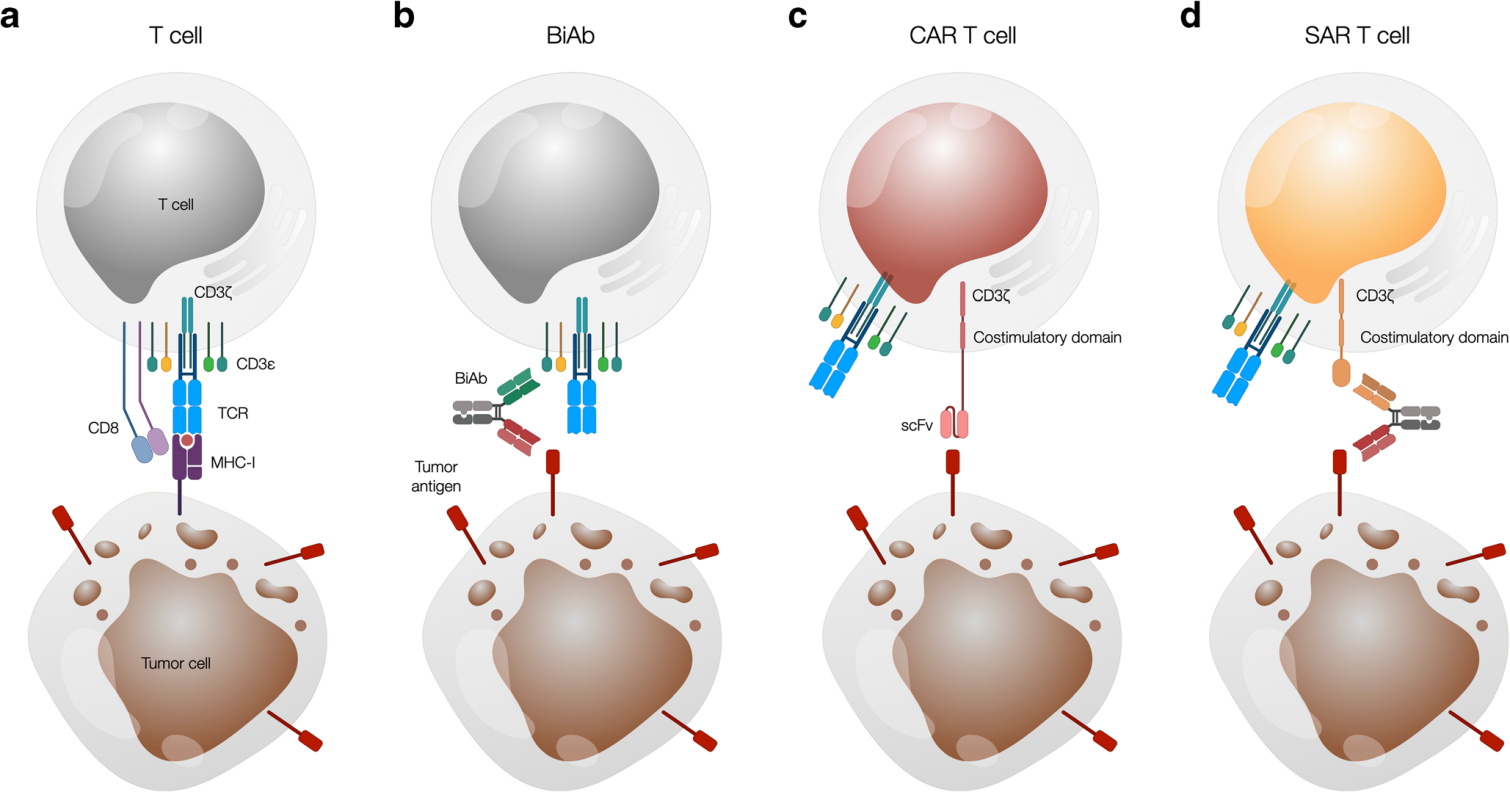
Schematic presentation of the interaction between T cells and tumor cells via a TCR, a BiAb, a CAR, and a SAR in combination with a BiAb. **a** A CD8^+^ T cell recognizes a tumor cell presenting a peptide from a tumor antigen on MHC class I via its TCR. **b** A BiAb mediates T cell recognition of a tumor cell by binding to both an antigen on the T cell surface, most commonly CD3, and a tumor cell surface antigen. **c** A T cell genetically modified to express a CAR binds a surface antigen expressed on the tumor cell via the scFv domain of the CAR in an MHC-independent manner. **d** A SAR-transduced T cell interacts with a tumor cell via a BiAb binding the SAR extracellular domain and a tumor cell surface antigen. BiAb, T cell redirecting bispecific antibody; CAR, chimeric antigen receptor; SAR, synthetic agonistic receptor; TCR, T cell receptor

BiAb-induced immune synapses formed between T cells and antigen-expressing target cells are very similar to the classical cytolytic immune synapse formed via the TCR-MHC interaction (Fig. 1a, b) [36]. In contrast, CAR T cells form an atypical synapse which is smaller and less organized and induces faster, stronger, and shorter signaling compared to the classical immune synapse (Fig. 1c). It also mediates faster target cell lysis by accelerated recruitment of lytic granules to the synapse and more rapid T cell detachment [37].

CAR T cells can kill antigen-expressing tumor cells via the release of cytotoxic granules containing perforin and granzymes, through the Fas-FasL pathway, and by sensitizing the tumor stroma following the release of pro-inflammatory cytokines [5•]. CAR activation was shown to upregulate FasL on T cells [38], and interferon-*γ* stimulation leads to Fas upregulation on some colon carcinoma cell lines and increased their susceptibility to CAR T cell–mediated killing [39]. BiAb are known to induce cytotoxicity via perforin and granzyme B [40]. Both BiAb and CAR T cells can mediate serial tumor cell killing [3••]. Interestingly, both strategies could mediate lysis of antigen-negative tumor cells that were in direct contact with antigen-positive cells, most likely involving the Fas-FasL axis in both cases [41, 42]. This suggests that Fas-FasL–based killing can also be mediated by BiAb.

### Antigen Spreading

Following antigen-specific tumor cell lysis, the released antigens may be taken up by dendritic cells and cross-presented to T cells, priming additional T cell responses in a process known as antigen or epitope spreading. There is evidence demonstrating that tumor-specific CD8^+^ T cells can mediate this process [43]. After treatment with mesothelin-specific CAR T cells, novel antibodies in two cancer patients could be detected using high-throughput serological analysis and immunoblotting. Both patients showed clinical antitumor activity following treatment despite not receiving lymphodepletion therapy before CAR T cell infusion [44]. Another study could show that clonal expansion of endogenous T cells could be induced by anti-mesothelin CAR T cells in several solid tumor patients, which was detected by deep sequencing of the TCR beta chain. This was not observed in patients receiving lymphodepletion prior to CAR T cell transfer [45]. Taken together, these studies show that CAR T cells can induce broadening of humoral responses as well as T cell epitope spreading in patients, effects that appear to be hampered by lymphodepletion. An example of epitope spreading has also been reported for BiAb therapy. A BiTE targeting Wilms’ tumor protein (WT1) led to the expansion of secondary T cell clones (with specificity for tumor-associated antigens other than WT1) in in vitro co-cultures of patient PBMCs with autologous tumor cells [46].

### CD4^+^/CD8^+^ T Cells and T Cell Phenotype

For both CAR T cells and BiAb, CD4^+^ T cells not only provide support for CD8^+^ T cells but have been shown to be directly cytotoxic [47•], although in a slower fashion. Further, CD4^+^ CAR T cells are less prone to activation-induced cell death [1•] and persist longer in vivo [48].

While less differentiated CAR T cells (naïve, stem cell memory, central memory) show better efficacy in vivo, it is mainly antigen-experienced T cells (effector memory) that mediate lysis via BiAb [2••, 47•, 49•]. Interestingly, BiAb have even been shown to redirect regulatory T cells to kill tumor cells [50].

### Manufacturing

One of the greatest differences between the two strategies is the manufacturing process. Thus far, CAR T cells have to be produced individually for each patient, a costly and laborious process (2 to 4 weeks) spanning lymphocyte apheresis to reinfusion, during which the disease may progress [49•]. After leukapheresis, patient T cells are isolated and activated before they are genetically modified with the CAR construct and expanded [51]. After quality testing, the product is shipped to the patient, who is pre-conditioned with lymphodepleting chemotherapy before CAR T cell infusion.

Lymphodepletion is not required prior to BiAb treatment. Additional obstacles for CAR T cell therapy include the challenge of achieving sufficient T cell numbers following leukapheresis and ex vivo expansion of the transduced T cells [52, 53].

In contrast, BiAb are off-the-shelf biologics that are easier to produce recombinantly and purify.

They bear the additional advantage of facile dose management, which is often challenging or not possible in the CAR T cell setting. However, based on the currently approved products, CAR T cells seem to be more efficacious than blinatumomab (see Table 1).

### T Cell Expansion and Persistence

Another major difference between CAR T cells and BiAb is the reliance on T cell expansion and persistence. While CAR T cells greatly rely on CAR T cell expansion, which can be higher than 1000-fold [54], T cell expansion is less important for BiAb because any antigen-experienced T cell can be engaged for tumor cell killing [47•]. With respect to recurrence after successful therapy, CAR T cells possess the advantage that they can engraft long term in the patient and thus attack recurring tumors, while BiAb action is abolished shortly after the last infusion [47•]. The impact of gene editing approaches on the production of a more refined CAR T cell product will broaden this disparity in years to come [55].

### Adverse Events

There are two main adverse events, one being CRS, a systemic response caused by antigen-specific T cell activation and subsequent release of pro-inflammatory cytokines. The other is neurotoxicity, otherwise referred to as immune effector cell–associated neurotoxicity syndrome (ICANS) [56]. CRS is generally more frequent and severe in CAR T cell therapy (see Table 1), often occurring in the first days after treatment and correlating with disease burden [3••, 57, 58]. CRS and ICANS are currently managed using an IL-6 receptor-blocking antibody (tocilizumab) and corticosteroids. To reduce these adverse events, pre-treatment with dexamethasone and step-up dosing have proven successful for blinatumomab, while split dosing has been tested in the CAR T cell setting [3••]. In addition, on-target off-tumor toxicities can be a major concern that depends on the expression profile of the targeted antigen in healthy tissues. In the case of B cell malignancies treated with anti-CD19 BiAb or CAR T cells, the consequent B cell aplasia has been largely manageable by the infusion of immunoglobulins [59, 60].

### Relapse

Despite high initial response rates, many patients relapse after anti-CD19 CAR T cell or blinatumomab treatment (see Table 1). However, the rate of CD19-negative relapses after initially successful therapy seems to be higher in CAR T cell–treated patients than in blinatumomab-treated patients (see Table 1). It is important to remember that blinatumomab is often used as a bridge to allogeneic stem cell transplantation. Such a transplantation would rather be the choice (if available) in case of relapse in spite of CAR T cell treatment [61, 62]. Along these lines, differences in antigen-loss variants might simply be a reflection of a lower treatment pressure with blinatumomab compared to CAR T cells [2••]. Many approaches that are currently in development aim to improve either therapy alone or combine the best of both approaches in efforts to develop novel solutions. These perspectives and their potential are discussed in the final section below.

*CR* complete remission, *CRh* CR with partial hematologic recovery, *CRi* CR with incomplete hematologic recovery

## Future Perspectives

Despite the high efficacy of CAR T cell and BiAb treatments, several hurdles continue to hamper their broader applicability. To tackle treatment-related toxicity, which has been especially problematic for CAR T cells (see Table 1), many approaches have been developed to improve their safety by making them more controllable (see Table 2). In addition, many CAR T cell– or BiAb-treated patients relapse due to antigen escape and, in the case of CAR T cells, limited persistence of the transferred T cells. This, alongside tumor antigen heterogeneity, has prompted the development of modular approaches combining T cells engineered with a CAR-like synthetic receptor and BiAb adapters targeting this receptor and a tumor antigen (see Table 2). These have the flexibility to redirect engineered T cells toward multiple targets [82].

**Table 2:** Limitations of CAR T cells and BiAb therapy and strategies to overcome them

	Strategy	Examples	Status
Improving controllability			
CAR T cells	Suicide receptor that is targetable by already approved monoclonal antibodies	CD20 [91, 92] Truncated EGFR [93]	In phase 1 clinical trial (NCT04318678) In phase 1 clinical trials (NCT01815749, NCT02051257)
	Suicide gene induced by small molecule	iCasp9 [94•]	Phase 1 clinical trials completed, but no results published yet (NCT02107963, NCT03958656), more phase 1 trials ongoing
		HSV thymidine kinase [95]	Preclinical results [95]
	Small molecule–controlled CAR expression/activity	CAR subunit dimerizing agent [96, 97] Dasatinib [98] SWIFF CAR [99] PROTAC compound [100]	In phase 1 clinical trial (NCT04650451) In phase 1 clinical trial (NCT04603872) Preclinical results [99] Preclinical results [100]
	Modular CAR platforms with bispecific adaptor molecule	UniCAR [83•] SUPRA CAR [84•] Switch CAR [85•] SAR [86•]	In phase 1 clinical trials (NCT04633148, NCT04230265) Preclinical results [84•] In phase 1 clinical trial (NCT04450069) Preclinical results [86•]
BiAb	Short half-life	Blinatumomab [10, 11]	FDA and EMA approved [65, 66]
	Dosing	Step-up dosing [57, 58]	Clinical application [101]
	Modular BiAb	UniMab [83•]	Preclinical results [83•]
Increasing T cell persistence			
CAR T cells	More naïve T cell subsets	Stem cell memory [102] Naïve, central memory [103]	In phase 1/2 clinical trial (NCT03288493) In phase 1 clinical trials (NCT02706405, NCT02146924)
	Using co-stimulatory domains favoring persistence	4-1BB [104, 105] ICOS [106] CD27 [107] Point-mutated CD28 [108]	FDA and EMA approval [25, 27] Preclinical results [106] Preclinical results [107] Preclinical results [108]
	Ratio CD4^+^ to CD8^+^ T cells	1:1 ratio [68]	Successful in phase 1/2 clinical trial (NCT01865617 [68, 109])
	Co-expression of 4-1BBL on CD28 CAR T cells	[105]	In phase 1 clinical trial (NCT03085173)
	Incorporating cytokine signaling	IL-2 receptor *β*-chain + STAT3-binding motif [110]	Preclinical results [110]
	Gene editing	Tet2 disruption [111]	Case report and preclinical results [111]
	Modular CAR platforms with bispecific adaptor molecule dosing to favor memory formation	[87•]	Preclinical results [87•]
	Combination with oncolytic virus, e.g., also expressing cytokines	Oncolytic virus expressing IL-15 & RANTES [112]	In phase 1 clinical trial (NCT03740256) (without cytokines)
Reducing on-target off-tumor effects			
CAR T cells	Affinity tuning	[24]	Preclinical results [24]
	Logic gating	Split CAR [113, 114] iCAR [115]	Preclinical results [113, 114] Preclinical results [115]
	Conditional CAR expression	SynNotch CAR [116] HIF-CAR [117]	Preclinical results [116] Preclinical results [117]
	Masking of antigen-binding site by peptide cleavable by tumor-associated protease	Masked CAR [118]	Preclinical results [118]
BiAb	Affinity tuning	[21, 119]	Preclinical results [21, 119]
	Split BiAb: CD3-binding site formed when both halves bind tumor antigens	Split BiAb [120•]	Preclinical results [120•]
	Masking of antigen-binding site by peptide cleavable by tumor-associated protease	Probody [121] Coiled-coil masking [122]	Preclinical results [121] Preclinical results [122]
Targeting antigen heterogeneity and antigen escape			
CAR T cells	Mixing multiple CAR T cell products	Anti-EGFR + anti-CD133 [123]	Preclinical results [123]
	Transduction of T cells with multiple CAR constructs	Anti-CD19 + anti-CD123 [124] Anti-HER2 + anti-IL-13Rα2 [125] Anti-BCMA + anti-CS1 [126]	Preclinical results [124] Preclinical results [125] In phase 1 clinical trial (NCT04156269)
	Bispecific (tandem) CAR constructs	Anti-HER2 + anti-IL-13Rα2 [127] Anti-CD19 + anti-CD22 [128] Anti-CD19 + anti-CD20 [129]	Preclinical results [127] Successful in case report [128], in phase 1 clinical trials (NCT04034446, NCT03919526) Successful in phase 1 and phase 1/2 clinical trials (NCT03019055 [129], NCT03097770 [130]), more phase 1 clinical trials ongoing
	Modular CAR platforms with multispecific adaptor molecules	Anti-CD33 + anti-CD123 [131]	Preclinical results [131]
BiAb	Combining multiple BiAb, multispecific BiAb	Anti-PSMA + anti-PSCA [132]	Preclinical results [132]
CAR T cells + BiAb	CAR T cells secreting BiAb	Anti-EGFRvIII CAR + anti-EGFR BiTE [88•]	Preclinical results [88•]
	CAR T cells + oncolytic virus secreting BiAb	Anti-FR-*α* CAR + anti-EGFR BiTE [133]	Preclinical results [133]
Increasing T cell infiltration			
CAR T cells	Expression of chemokine receptors	CCR4 [134] CCR2b [135] CXCR2 [136]	In phase 1 clinical trial (with anti-CD30 CAR T cells) (NCT03602157) Preclinical results [135] Preclinical results [136]
	CAR targeting tumor stroma	Anti-FAP CAR [137]	In phase 1 clinical trial (NCT03932565)
	Expression of extracellular matrix-degrading enzymes	Heparanase [138]	Preclinical results [138]
	Expression of cytokines	IL-7 and CCL19 [139]	In phase 1 clinical trials (NCT03932565, NCT04381741)
	Combination with oncolytic virus, e.g., also expressing cytokines	Oncolytic virus expressing IL-15 and RANTES [112] Oncolytic virus expressing IL-2 and TNF-*α* [140]	In phase 1 clinical trial (NCT03740256) (without cytokines)
BiAb	Combination with oncolytic virus	[141]	Preclinical results [141]
Counteracting immunosuppression			
CAR T cells	Combination with checkpoint-blocking antibodies	Anti-PD-1 [142] Anti-PD-L1 [143]	Successful in phase 1 clinical trials (ChiCTR-ONN-16009862/ChiCTR1800019288 [142], NCT03726515), more phase 1 clinical trials ongoing Successful in phase 1 clinical trial (NCT02926833) [143], more phase 1 and phase 1/2 clinical trials ongoing
	Gene silencing of inhibitory receptors	PD-1 [144] Fas [145] A_2A_R [146]	In phase 1 clinical trials (NCT03545815, NCT04213469) Preclinical results [145] Preclinical results [146]
	Co-transduction with dominant-negative decoy receptors (DNR)	TGF-*β* DNR [147] PD-1 DNR [148] Fas DNR [149]	In phase 1 clinical trials (NCT03089203, NCT04227275) In phase 1 clinical trial (NCT04577326) Preclinical results [149]
	Co-transduction with switch receptor	PD-1-CD28 [150] IL-4R-IL-7R [151]	In phase 1 clinical trials (NCT02937844, NCT03932955) Preclinical results [151]
	CAR T cells secreting checkpoint-blocking antibodies	Anti-PD-L1 [152] Anti-PD-1 [153]	In phase 1 clinical trial (NCT04556669) In phase 1 clinical trials (NCT04489862, NCT03182803)
	CAR T cells expressing cytokines (TRUCK)	IL-12 [154] IL-15 [155] IL-18 [156]	In phase 1 clinical trials (NCT03542799, NCT02498912) In phase 1 clinical trials (NCT04377932, NCT04715191) In phase 1 clinical trial (NCT04684563)
	Combination with an oncolytic virus expressing checkpoint-blocking antibody	Oncolytic virus expressing PD-L1 blocking mini-body [157]	Preclinical results [157]
BiAb	Combination with checkpoint blockade	Anti-PD-1/anti-PD-L1/anti-CTLA-4 [158, 159]	In phase 1 clinical trials (NCT02879695, NCT03792841)
	Combination with bispecific 4-1BB agonists	4-1BBL-anti-FAP + anti-CD3-anti-CEA 4-1BBL-anti-CD19 + anti-CD3-anti-CD20 [160•]	Preclinical results [160•]
	Trispecific antibody targeting CD3, tumor antigen, and checkpoint molecule	CiTE [90•]	Preclinical results [90•]

Among these platforms are the universal CAR (UniCAR) [83•], split universal and programmable (SUPRA) CAR [84•], switch CAR [85•], and the synthetic agonistic receptor (SAR) developed by our lab (Fig. 1d) [86•]. The activity of the modular CAR T cell can be controlled by the affinities of the two binding sites, as well as the half-life and dosing of the BiAb to limit side effects while retaining antitumor efficacy.

In addition, multiple tumor antigens can be simultaneously or sequentially targeted to address antigen heterogeneity and reduce antigen escape [82]. Moreover, by administering decoys for the CAR adaptors, their activity can be controlled even more tightly [84•]. Interestingly, Viaud et al. could enhance memory T cell formation by including “rest” phases between dosing cycles of the CAR adapter [87•]. It is important to note that while advantageous in terms of controllability, short half-life formats of BiAb mean that regular infusions will be required. Combining CAR T cells and BiAb will likely present hurdles in the form of practicality and cost. Therefore, CAR adaptors will most practically be useful in the context of an “off-the-shelf” universal allogeneic CAR T cell line that can be combined with different adaptors for different tumor antigens.

Translating the success of BiAb and CAR T cell therapies to solid cancer indications poses additional challenges. As a result, attempts to improve T cell recruitment into the tumor render T cells more resistant to the immunosuppressive tumor microenvironment and target antigen heterogeneity among tumor cells are currently underway (see Table 2). One noteworthy strategy presented by Choi and colleagues employs engineered CAR T cells to secrete BiAb targeting a second tumor antigen to treat glioblastoma. They could show this to be a promising approach in a mouse model which shows antigen-negative relapse when CAR T cells alone are employed [88•]. Trafficking of CAR T cells may be enhanced by equipping them with, e.g., chemokine receptors for chemokines expressed in the tumor [89•]. Trispecific antibodies targeting CD3, a tumor antigen, and a checkpoint molecule have been shown to counteract immunosuppression [90•].

Table 2 provides an overview of the current strategies being developed to overcome the aforementioned challenges of CAR T cells and BiAb.

## Conclusion

Despite the apparent overlap between CAR T cell and BiAb approaches (such as their application to target the same antigen for some of the same indications), it remains clear that both therapies offer distinct benefits. The emergence of treatments that combine the best of both the CAR and BiAb worlds highlights this, as shown by SAR T cells that utilize BiAb to enable selective and modular control over T cell activation.

Nevertheless, both CAR and BiAb approaches continue to be developed in their own right, with advancements addressing the shortcomings of either approach. Combining BiAb with bispecific 4-1BB agonists is one such example, where the lack of a co-stimulatory signal 2 is effectively overcome. For CAR T cells, various approaches have been developed by either limiting their activation to the tumor microenvironment, like the hypoxia-inducible factor (HIF) or synthetic Notch (SynNotch) CAR, or by making their activation more controllable from the outside, e.g., by administering small molecules or antibodies to activate or inhibit CAR T cell activity.

Due to the speed at which both therapies have gained regulatory approval, mechanistic insights into the drivers of treatment efficacy, disease relapse, and treatment-related toxicities are only now being uncovered. Translating these insights from bench to bedside in a timely and effective manner will be important to achieve greater patient benefit.

## Abbreviations

A_2A_: Adenosine 2A Receptor
BCMA: B Cell Maturation Antigen
CCL: C-C Motif Chemokine Ligand
CCR: C-C Motif Chemokine Receptor
CEA: Carcinoembryonic Antigen
CiTE: Checkpoint inhibitory T cell-Engaging
CTLA-4: Cytotoxic T Lymphocyte Associated Protein 4
CXCR: C-X-C Motif Chemokine Receptor
DNR: Double-Negative Receptor
EGFR: Epidermal Growth Factor Receptor
EGFRvIII: EGFR Variant 3
FR-α: Folate Receptor α
HER2: Human Epidermal Growth Factor Receptor 2
HIF: Hypoxia-Inducible Factor
HSV: Herpes Simplex Virus
iCAR: inhibitory CAR
iCasp9: inducible Caspase 9
ICOS: Inducible T Cell Costimulatory
PD-1: Programmed Cell Death Protein 1
PD-L1: Programmed Cell Death 1 Ligand 1
PROTAC: Proteolysis-Targeting Chimaera
PSCA: Prostate Stem Cell Antigen
PSMA: Prostate Specific Membrane Antigen
RANTES: Regulated upon Activation, Normal T cell Expressed and Presumably Secreted
STAT3: Signal Transducer and Activator of Transcription 3
SWIFF: Switch-Off CAR
SynNotch: Synthetic Notch
TET2: Tet Methylcytosine Dioxygenase 2
TGF-β: Transforming Growth Factor β
TNF: Tumor Necrosis Factor
TRUCK: T Cells Redirected for Antigen-Unrestricted Cytokine-Initiated Killing
